# Leaf Ontogeny Shapes Divergent Physiological and Metabolic Responses to Contrasting Nitrogen Forms in Chinese Fir (*Cunninghamia lanceolata* (Lamb.) Hook)

**DOI:** 10.3390/ijms27093789

**Published:** 2026-04-24

**Authors:** Wen-Yang Fu, Ya-Li Zhang, Wan-Ting Yu, Zhong-Wei Zhang, Shu Yuan, Guang-Deng Chen, Jian Zeng

**Affiliations:** International Science and Technology Cooperation Base for Efficient Utilization of Nutrient Resources and Fertilizer Innovation, College of Resources, Sichuan Agricultural University, Chengdu 611130, China; fwyang_08@163.com (W.-Y.F.); yilia18783022120@163.com (Y.-L.Z.); yuwanting0209@163.com (W.-T.Y.); zzwzhang@126.com (Z.-W.Z.); roundtree@sicau.edu.cn (S.Y.); gdchen@sicau.edu.cn (G.-D.C.)

**Keywords:** Chinese fir, leaf ontogeny, N forms, physiological response, metabolic reprogramming, N deposition

## Abstract

Atmospheric nitrogen (N) deposition is altering global forest ecosystems, with nitrate rising to rival ammonium as a dominant N form, yet how leaf ontogeny orchestrates carbon–nitrogen (C-N) metabolic coordination under contrasting N forms remains poorly understood. We conducted a field experiment investigating the physiological and metabolic responses of young and old leaves of Chinese fir (*Cunninghamia lanceolata*) to ammonium and nitrate addition. Young leaves, functioning as active sinks, exhibited enhanced photosynthetic performance and growth-oriented N assimilation under N addition, with disproportionately stronger responses to nitrate. In contrast, old leaves, acting as source tissues, showed limited photosynthetic plasticity but accumulated higher non-structural carbohydrates and elevated N assimilation enzyme activities, particularly under nitrate addition. Phytohormone profiles supported this ontogenetic divergence, with young leaves showing higher auxin levels while old leaves exhibited increased abscisic acid and salicylic acid contents. Metabolomic analysis further revealed age-dependent reprogramming of amino acid metabolism, identifying key metabolites coordinating C-N balance. These findings demonstrate a leaf ontogeny-mediated spatial division of metabolic labor in Chinese fir, wherein old leaves function as metabolic buffers stabilizing whole-plant C-N homeostasis under fluctuating N supply, providing new insights into plantation responses to contrasting N deposition regimes.

## 1. Introduction

Global terrestrial ecosystems are experiencing unprecedented alterations in nitrogen (N) deposition driven predominantly by agricultural intensification and industrial processes [1]. Atmospheric N enters natural and seminatural ecosystems through precipitation and dry deposition, modulating plant growth via nutritional status and photosynthetic processes [2,3,4]. In China, atmospheric N deposition has risen sharply since the 1980s and subsequently stabilized at elevated levels, with a fundamental qualitative shift from ammonium (NH_4_^+^) dominance toward increasing co-dominance with nitrate (NO_3_^−^) [5]. Because the assimilation of NH_4_^+^ and NO_3_^−^ entails distinct energetic costs, subcellular localization, and enzymatic pathways, this shifting N composition poses novel challenges to carbon–nitrogen (C-N) biogeochemical cycling and plant physiological acclimation in forest ecosystems. N is an indispensable macronutrient for the formation of proteins, nucleic acids, chlorophyll, and other bioactive molecules [6], yet the ecological and physiological consequences of contrasting N forms on plant C-N coordination remain largely unresolved.

To optimize growth under fluctuating N environments, plants must tightly coordinate C and N metabolism. This metabolic coordination, however, is not uniform across the plant body, but is strongly shaped by leaf ontogeny, which dictates whole-plant sink–source dynamics. Young, expanding leaves typically function as active sinks, demanding substantial C and N resources to fuel structural construction and photosynthetic machinery assembly. In contrast, older leaves act primarily as source tissues, exporting photoassimilates and serving as nutrient storage and buffering pools [7,8]. Leaf functional traits are known to be sensitive to N availability, especially in ecosystems with low N deposition backgrounds [9,10], and studies on *Arabidopsis* and wild soybean have highlighted the differential responses of young and old leaves to N availability and environmental stress [11,12]. Despite the critical importance of these developmental stages in shaping leaf functional traits [13], current research frequently treats the forest canopy as a physiologically homogeneous entity. How leaf ontogeny orchestrates C-N metabolic reprogramming and resource partitioning under contrasting N forms remains a critical knowledge gap, particularly in long-lived woody species that rely heavily on complex resource reallocation for sustained productivity.

The spatiotemporal division of metabolic labor between different-aged leaves is strictly modulated by complex phytohormonal networks. Phytohormones act as central hubs integrating environmental nutrient signals with intrinsic developmental programs, regulating plant growth and development while mediating responses to environmental changes and influencing C and N assimilation [14,15]. Among these hormones, cytokinin (CK) is a key regulator of cell division, shoot development, delayed leaf senescence, and photosynthetic function, and also plays an important role in plant responses to nitrogen availability. Previous studies have shown that nitrate can regulate cytokinin biosynthesis and metabolism, thereby linking external N signals with developmental regulation [16,17]. The hormonal profiles vary markedly across leaf developmental stages: auxin (IAA) is indispensable for cell expansion and vascular development in young sink tissues, whereas abscisic acid (ABA) and salicylic acid (SA) are frequently implicated in stress acclimation, stomatal regulation, and the remobilization of reserves in mature tissues [18,19]. CK also shows developmental regulation and generally contributes to the maintenance of leaf growth and delayed senescence, and may therefore participate in the functional differentiation between younger and older leaves under contrasting N conditions. Phytohormones also exert feedback regulation on N metabolism, aligning plant nutrition with developmental stage [20]. Investigating the interplay between these hormonal signals and primary C-N metabolites across distinct leaf developmental stages is therefore essential for deciphering the mechanistic basis of plant adaptation to contrasting N forms.

*Cunninghamia lanceolata* (Lamb.) Hook (Chinese fir) is a fast-growing, ecologically significant timber species widely distributed in subtropical China [21,22]. The productivity decline associated with successive monoculture planting has become an increasingly prominent issue [23], underscoring the urgent need for optimized, form-specific nutrient management strategies. As N plays a critical role in sustaining plantation productivity by affecting both soil-level processes and leaf-level functions, understanding how contrasting N forms interact with leaf developmental stage to shape C-N metabolic strategies is particularly important in this species. Previous studies have shown that Chinese fir exhibits age-related variation in N acquisition strategies and contrasting preferences for NH_4_^+^ and NO_3_^−^ during root development [24,25], indicating that developmental stage and N form jointly influence nutrient-use characteristics in this species. However, these studies have mainly focused on belowground N uptake traits, whereas how different leaf ages coordinate physiological and metabolic adjustment to contrasting N forms remains poorly understood.

To address these knowledge gaps, we conducted a field-controlled N addition experiment simulating ammonium and nitrate enrichment, with the following objectives: (1) to characterize the functional responses of young and old leaves to ammonium and nitrate addition; (2) to clarify the regulatory effects of N forms on leaf functional traits across developmental stages; and (3) to uncover the age-dependent nutrient strategies underpinning whole-plant C-N coordination. By integrating physiological assessments with targeted metabolomics, this study provides new insight into how different leaf ages of Chinese fir adjust physiological and metabolic processes in response to contrasting N forms under increasing atmospheric N deposition, and provides a reference for further studies on nutrient regulation in plantation trees.

## 2. Results

### 2.1. Photosynthetic Performance and C Metabolism Under Contrasting N Forms Addition

**Figure 1:**
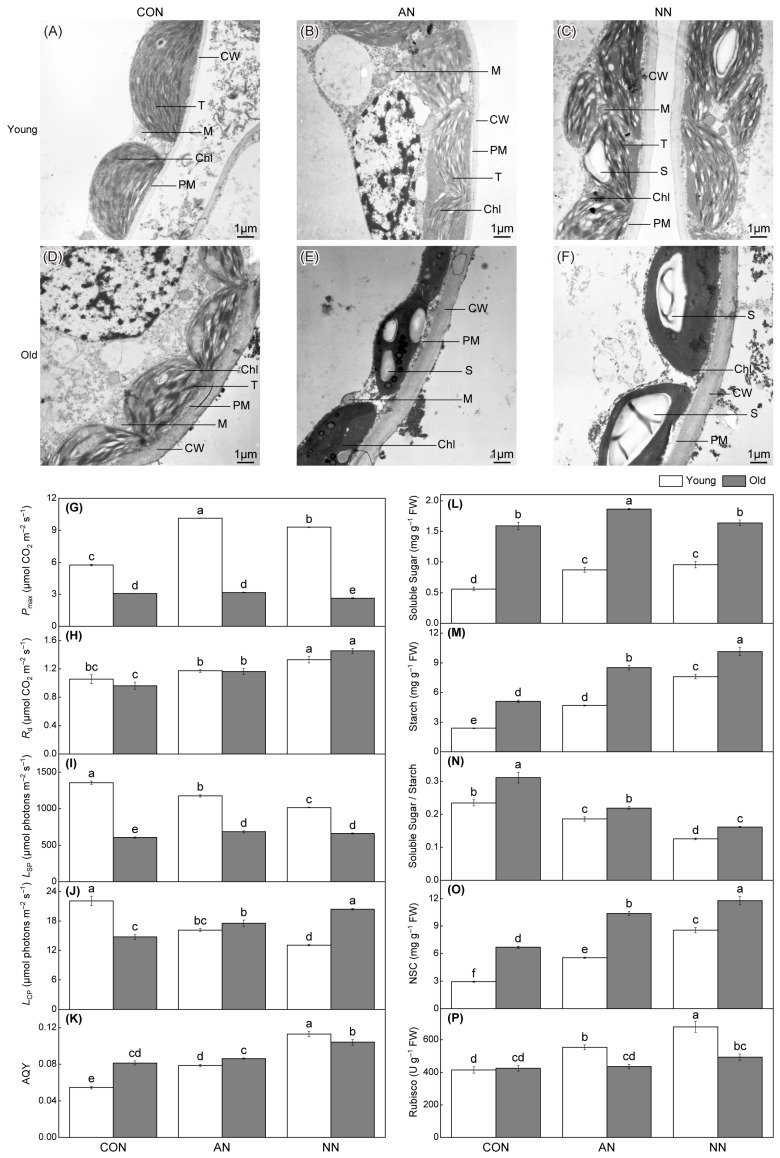
Differential effects of contrasting N forms addition on photosynthetic potential in young and old leaves of Chinese fir. Transmission electron microscopy images of chloroplast ultrastructure in young leaves (**A**–**C**) and old leaves (**D**–**F**) under control without N addition (CON), NH_4_^+^-N addition (AN) and NO_3_^−^-N addition (NN). Photosynthetic parameters and carbohydrate-related traits in young (white bars) and old leaves (gray bars), including *P*_max_, maximum net photosynthetic rate (**G**); *R*_d_, dark respiration rate (**H**); *L*_SP_, light saturation point (**I**); *L*_CP_, light compensation point (**J**); AQY, apparent quantum yield (**K**); soluble sugar content (**L**); starch content (**M**), soluble sugar/starch ratio (**N**); NSC, non-structural carbohydrate content (**O**); Rubisco activity (**P**). Different lowercase letters indicated significant differences among treatments and between leaf ages (*p* < 0.05). Scale bars = 1 μm. CW, cell wall; PM, plasma membrane; Chl, chloroplast; M, mitochondria; T, thylakoid; S, starch granule.

Distinct differences in chloroplast ultrastructure were observed between young and old leaves of Chinese fir under different N sources (Figure 1). In young leaves, chloroplasts were predominantly spindle-shaped and closely associated with the cell wall, with orderly arranged thylakoid structures and tightly packed stromal lamellae. NO_3_^−^-N promoted greater starch accumulation than NH_4_^+^-N (Figure 1A–C). In old leaves, chloroplast ultrastructure varied among N treatments, characterized by cell wall thickening, granum disintegration, peripheral reticulum roughening, and starch accumulation, with NH_4_^+^-N notably inducing starch granule dilation (Figure 1D–F).

Photosynthetic performance and associated C metabolism differed markedly between leaf ages (Figure 1G–K). Maximum net photosynthetic rate (*P*_max_) and light saturation point (*L*_SP_) were consistently higher in young leaves. N addition enhanced *P*_max_ in young leaves but reduced it in old leaves. Dark respiration rate (*R*_d_) and apparent quantum yield (AQY) increased in both leaf types following N addition. Light compensation point (*L*_CP_) and *L*_SP_ decreased in young leaves but increased in old leaves under N addition, though *L*_SP_ remained higher in young leaves overall.

Carbohydrate accumulation and allocation also differed between leaf ages (Figure 1L–P). Old leaves contained higher soluble sugar, starch and non-structural carbohydrates (NSC) contents. N addition increased carbohydrate levels were observed in both ages, but reduced the soluble sugar/starch ratio; NO_3_^−^-N resulted in greater carbohydrate accumulation than NH_4_^+^-N, with the opposite trend for the sugar/starch ratio. Rubisco activity increased significantly in young leaves under N addition, particularly with NO_3_^−^-N, but showed little change in old leaves.

### 2.2. N Metabolism Under Contrasting N Forms Addition

**Figure 2:**
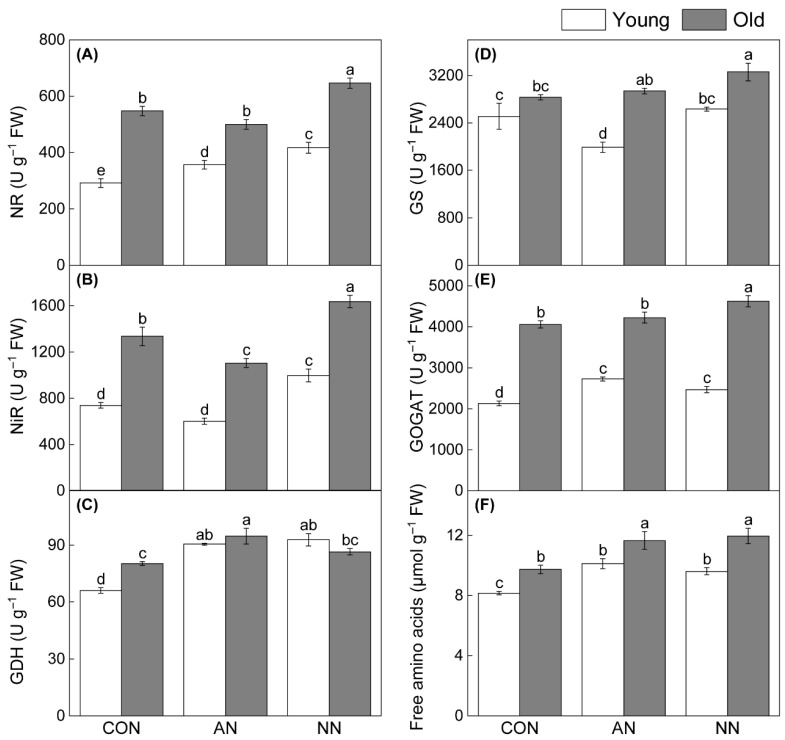
Effects of contrasting N forms addition on N metabolism in young and old leaves of Chinese fir. Nitrate reductase activity (**A**), nitrite reductase activity (**B**), glutamate dehydrogenase activity (**C**), glutamine synthetase activity (**D**), glutamate synthase activity (**E**), free amino acid content (**F**). Different lowercase letters indicated significant differences among treatments and between leaf ages (*p* < 0.05).

N metabolism-related enzyme activities and free amino acid content generally increased under N addition, except for glutamine synthetase (GS) activity in young leaves (Figure 2). Activities of nitrate reductase (NR), nitrite reductase (NiR), glutamate synthase (GOGAT), GS, and glutamate dehydrogenase (GDH) were significantly higher in old leaves than in young leaves, and free amino acid content followed the same pattern regardless of N form. Compared with NH_4_^+^-N addition, NO_3_^−^-N addition significantly enhanced NR and NiR activities (Figure 2A,B), while GS, GOGAT, and GDH activities responded differently between leaf ages. No significant difference in free amino acid content was detected between N forms in either leaf age (Figure 2F).

### 2.3. Phytohormone Profiling Under Contrasting N Forms Addition

**Figure 3:**
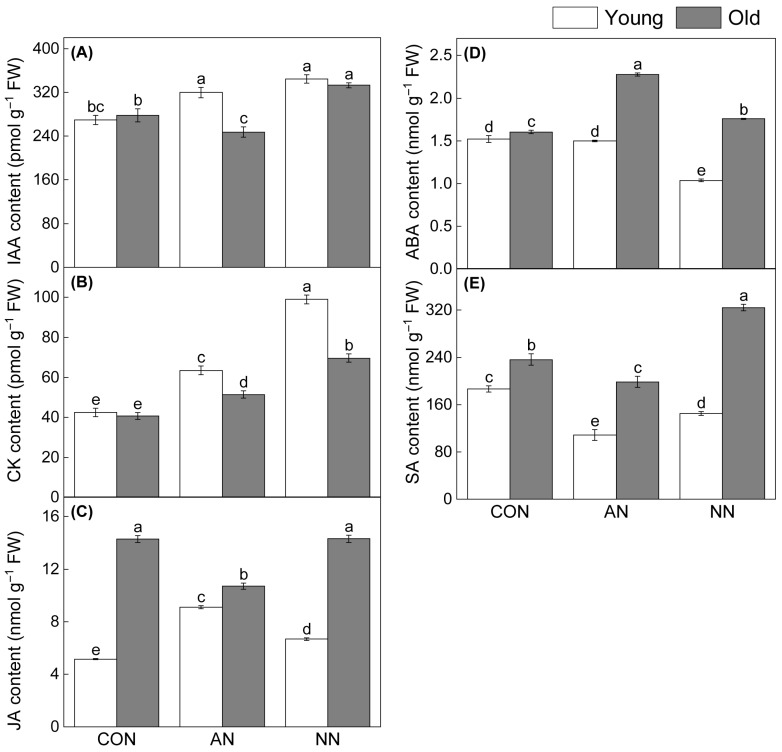
Effects of contrasting N forms addition on phytohormone contents in young and old leaves of Chinese fir. Indole-3-acetic acid (IAA) content (**A**), cytokinin (CK) content (**B**), jasmonic acid (JA) content (**C**), abscisic acid (ABA) content (**D**), and salicylic acid (SA) content (**E**). Different lowercase letters indicated significant differences among treatments and between leaf ages (*p* < 0.05).

Phytohormone contents varied significantly between leaf ages in response to different N forms (Figure 3). Jasmonic acid (JA), abscisic acid (ABA), and salicylic acid (SA) were consistently higher in old leaves, while indoleacetic acid (IAA) showed no consistent age-dependent pattern. In young leaves, N addition significantly increased IAA, CK, and JA but decreased ABA and SA relative to the control. In old leaves, NH_4_^+^-N addition significantly decreased IAA, JA and SA contents but increased ABA and CK, whereas NO_3_^−^-N significantly elevated IAA, CK, ABA and SA (Figure 3A,B,D,E). Compared with NH_4_^+^-N, NO_3_^−^-N significantly increased CK and SA while decreased ABA in both leaf ages. IAA was significantly elevated by NO_3_^−^-N only in old leaves (Figure 3). Notably, NO_3_^−^-N strongly amplified age-dependent differences in CK and SA accumulation, whereas NH_4_^+^-N had a weaker effect in old leaves.

### 2.4. Metabolomic Profiling Under Contrasting N Forms Addition

**Figure 4:**
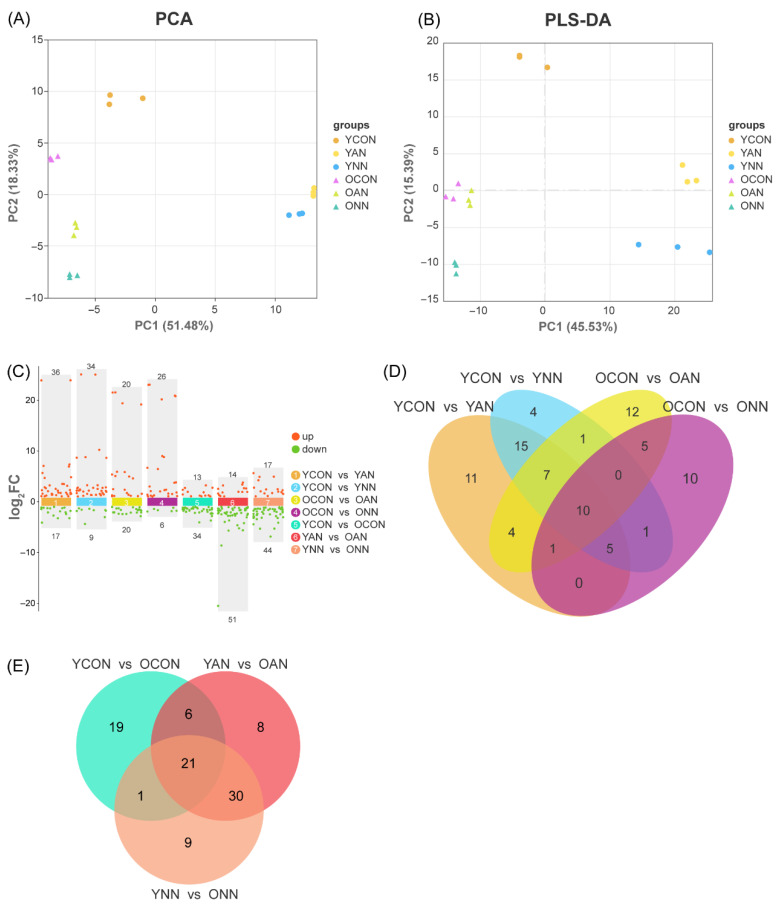
Multivariate analysis of metabolomics data and identification of differentially accumulated metabolites (DAMs). Principal component analysis (PCA) score plot of metabolome data (**A**), partial least squares-discriminant analysis (PLS-DA) score plot (**B**), distribution of DAMs identified in pairwise comparisons (**C**), Venn diagrams illustrated the shared and unique DAMs under differential N forms addition (**D**). Venn diagrams illustrated the shared and unique DAMs between different leaf types (**E**). YCON, young leaves under control conditions without N addition; YAN, young leaves under NH_4_^+^-N addition; YNN, young leaves under NO_3_^−^-N addition; OCON, old leaves under control conditions without N addition; OAN, old leaves under NH_4_^+^-N addition; ONN, old leaves under NO_3_^−^-N addition.

To characterize metabolic responses to contrasting N forms across leaf ages, metabolomic profiling was conducted (Figure 4). Principal component analysis (PCA) and partial least squares-discriminant analysis (PLS-DA) revealed clear separation by leaf age and N form, with PC1 explaining 51.48% of total variance and primarily reflecting leaf ontogeny (Figure 4A,B). A total of 562 metabolites were detected. Compared with control, 53 differentially accumulated metabolites (DAMs) (36 up-, 17 down-regulated) were identified in young leaves under NH_4_^+^-N addition (YAN), 43 DAMs (34 up-, 9 down-regulated) in young leaves under NO_3_^−^-N addition (YNN), 40 DAMs (20 up-, 20 down-regulated) in old leaves under NH_4_^+^-N addition (OAN), and 32 DAMs (26 up-, 6 down-regulated) in old leaves under NO_3_^−^-N addition (ONN) (Figure 4C).

DAMs were primarily enriched in pathways related to C and N metabolism, including amino acid metabolism, translation, membrane transport, and secondary metabolite biosynthesis (Figure S1). Age-dependent enrichment patterns were evident: young leaves showed preferential enrichment in amino acid biosynthesis and carbon metabolism, particularly aminoacyl-tRNA and glucosinolate pathways (Figure S1A,B); in old leaves, NH_4_^+^-N addition produced limited enrichment largely confined to amino acid and nucleotide metabolism (Figure S1C), while NO_3_^−^-N addition induced broader metabolic adjustments, particularly in secondary metabolism, with higher enrichment in anthocyanin biosynthesis (Figure S1D). NH_4_^+^-N specifically enriched ABC transporters and monobactam biosynthesis (Figure S1E), whereas NO_3_^−^-N uniquely enriched carbapenem biosynthesis and 2-oxocarboxylic acid metabolism in old leaves (Figure S1F).

N addition generated 15 unique DAMs in young leaves and 22 in old leaves relative to the control (Figure 4D,E). Unique DAMs in young leaves were mainly flavonoids, organic acids, and plant hormones, while old leaves showed a more diverse profile, additionally including amino acids. Old leaves also exhibited 17 unique DAMs relative to young leaves under N addition, NH_4_^+^-N addition, enriched fatty acids, amino acids, and flavonoids, while NO_3_^−^-N uniquely accumulated nucleotides and their derivatives (Tables S1 and S2).

### 2.5. DAMs Clustering and Metabolic Pathway Responses Under Contrasting N Forms Addition

**Figure 5:**
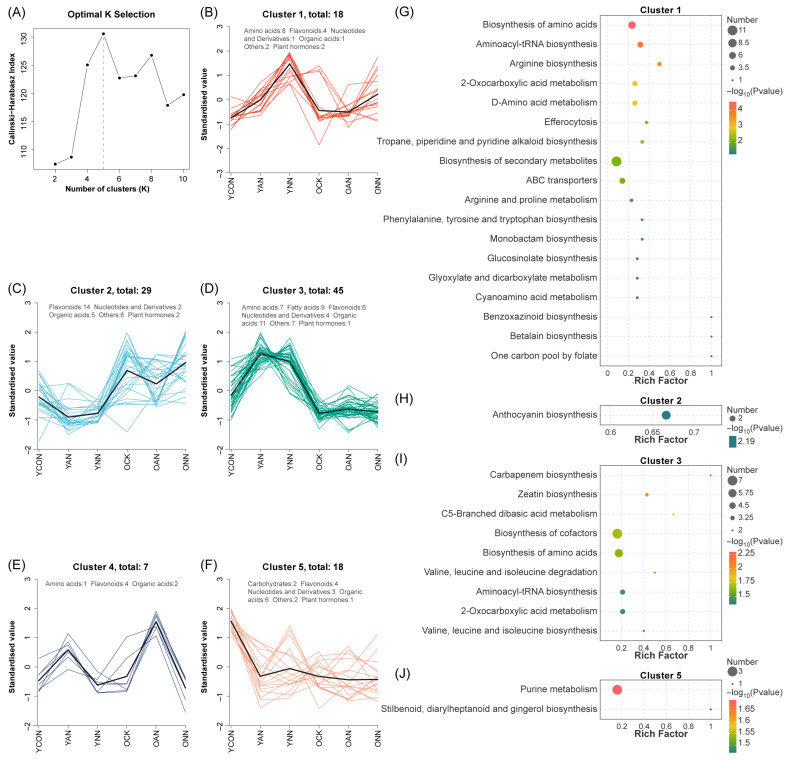
Metabolic clustering patterns and functional associations of differential accumulated metabolites (DAMs) in young and old leaves of Chinese fir. Determination of the optimal number of clusters for K-means clustering (**A**). K-means clustering profiles of DAMs across treatment and leave ages (**B**–**F**). Colored lines represented individual metabolites, and black lines indicated the average trend for each subclass. KEGG pathway enrichment analysis of DAMs in K-means Clusters 1, 2, 3, and 5. The rich factor represented the ratio of the number of metabolites mapped to a given pathway to the total number of annotated metabolites (**G**–**J**). Bubble size indicated the number of metabolites enriched in each pathway, and bubble color reflected the enrichment significance level.

K-means clustering identified five distinct DAM clusters across N form treatments and leaf ages (Figure 5A–F). Cluster 1 was specifically upregulated in young leaves under NO_3_^−^-N addition, while Cluster 3 increased in N-treated young leaves but decreased in old leaves; other clusters displayed treatment- or age-specific patterns.

KEGG (Kyoto Encyclopedia of Genes and Genomes) enrichment analysis revealed functional differentiation among clusters (Figure 5G–J). Clusters 1 and 3 were both enriched in amino acid-related pathways, including amino acid biosynthesis, aminoacyl-tRNA biosynthesis, and 2-oxocarboxylic acid metabolism, with Cluster 1 additionally enriched in ABC transporters and glucosinolate biosynthesis and Cluster 3 characterized by cofactor biosynthesis. Cluster 2 and Cluster 5 were specifically enriched in anthocyanin biosynthesis and purine metabolism, respectively, and no significant pathways were detected in Cluster 4.

**Figure 6:**
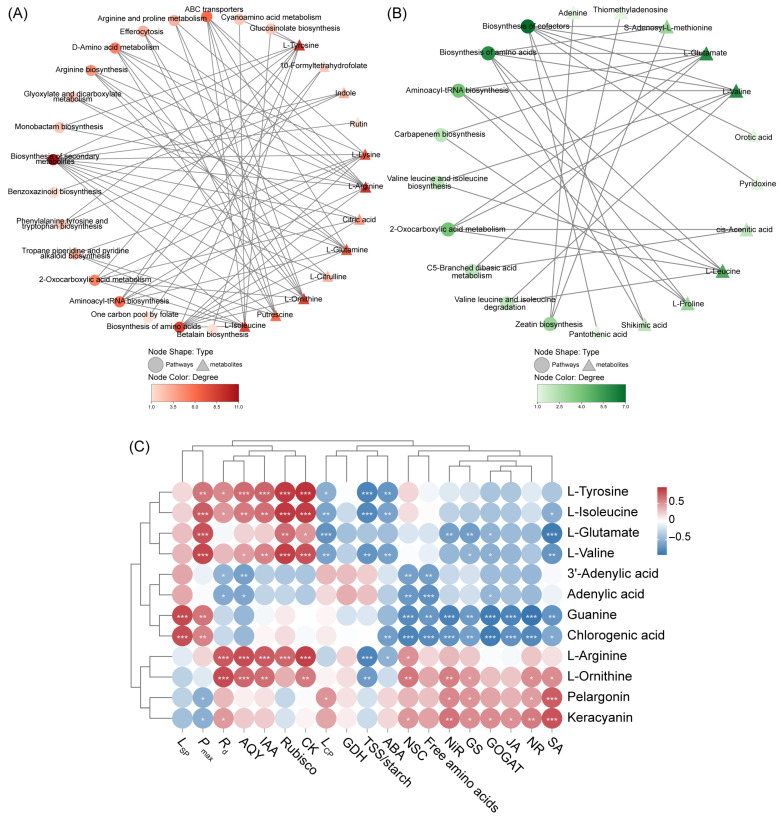
Pathway–metabolite network and correlation analyses of key differentially accumulated metabolites (DAMs) in young and old leaves of Chinese fir. Pathway–metabolite network analyses of enriched metabolic pathways in K-means Cluster 1 (**A**) and Cluster 3 (**B**). Circular and triangular nodes represented metabolic pathways and metabolites, respectively. Node color intensity corresponded to degree values, with darker colors indicating higher connectivity. Pearson correlation analysis between key DAMs and physiological indicators (**C**). Circle color represented the strength and direction of correlations (red, positive; blue, negative), and asterisks denoted statistical significance (*, *p* < 0.05; **, *p* < 0.01; ***, *p* < 0.001).

Network analysis identified aminoacyl-tRNA biosynthesis and ABC transporters as hub pathways in Cluster 1, with L-arginine, L-tyrosine, L-ornithine, and L-isoleucine showing high connectivity (Figure 6A); cofactor biosynthesis served as the central hub in Cluster 3, with L-glutamate and L-valine as key nodes (Figure 6B). Based on network topology and biological relevance, representative metabolites with relatively high connectivity were retained as key candidates. Representative metabolites from Clusters 2 and 5, including pelargonin, keracyanin, 3′-adenylic acid, adenylic acid, guanine, and chlorogenic acid, were also retained. The 12 key metabolites integrated into a heat map showed significant associations with multiple C- and N-related physiological parameters (Figure 6C), linking metabolic shifts to C-N regulation under contrasting N forms.

**Figure 7:**
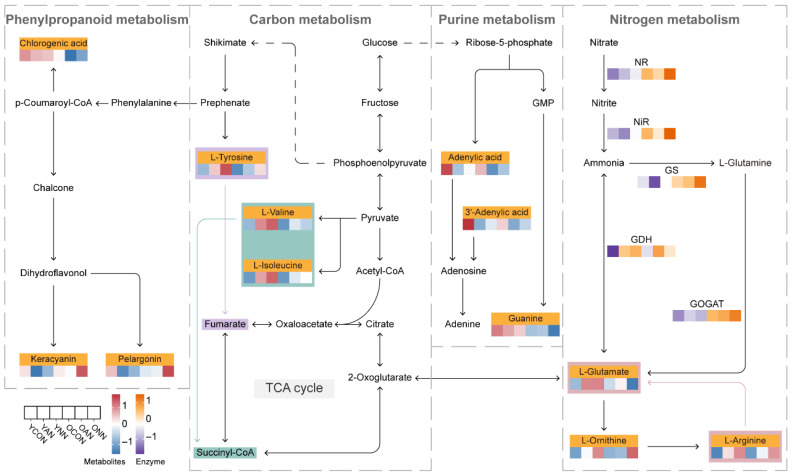
Schematic diagrams of key metabolites involved in N assimilation and amino acid biosynthesis in young and old leaves of Chinese fir under contrasting N form addition. Heat maps were generated based on row-wise z-score standardized values. Red or blue indicates the mean metabolite abundance, while orange or purple represents the enzyme activity. Solid arrows indicated relatively direct relationships based on KEGG pathway annotations, whereas dashed arrows indicated indirect relationships in which intermediate steps were omitted for schematic clarity.

Mapping these metabolites onto a reconstructed pathway network clarified age-dependent metabolic reprogramming (Figure 7). In young leaves, branched-chain and aromatic amino acids (L-tyrosine, L-valine and L-isoleucine) were preferentially upregulated, with stronger accumulation under NO_3_^−^-N addition than NH_4_^+^-N. In old leaves, N-allocation-associated amino acids (L-glutamate, L-arginine and L-ornithine) were enhanced, consistent with elevated N assimilation enzyme activities, particularly under NO_3_^−^-N addition. Purine metabolism showed a contrasting pattern, with nucleotide-related metabolites (3′-adenylic acid, adenylic acid, guanine) declining under N addition, particularly in old leaves. Phenylpropanoid and anthocyanin metabolites were more substantially reconfigured in old leaves, with NO_3_^−^-N exerting a stronger effect on anthocyanin-related compounds.

## 3. Discussion

Leaf functional traits vary with age and position, influencing substance and energy exchange between plants and their environment as well as plant survival strategies under changing conditions [26]. Sensing nutritional status and coordinating signal transduction within and between organs is essential for dynamically regulating C and N partitioning and utilization [27]. This study integrates physiological and metabolomic data in Chinese fir to characterize leaf age-dependent responses to contrasting N forms addition.

### 3.1. C and N Metabolism Variation in Young and Old Leaves

Plant organs dynamically adjust morphology and function in response to developmental stage, which in turn shapes their nutritional demands [28]. In this study, leaf age drove profound divergence in photosynthetic C metabolism, N assimilation, phytohormone profiles, and metabolite reprogramming under ammonium and nitrate addition, with nitrate generally exerting stronger regulatory effects than ammonium. These findings highlight an age-dependent C-N coupling mechanism mediating adaptive responses to contrasting N forms in Chinese fir.

Young leaves maintained intact chloroplast ultrastructure, higher photosynthetic capacity, and enhanced Rubisco activity under N addition, consistent with their role as the primary site for C fixation and growth. By contrast, old leaves exhibited deteriorated thylakoid structure, reduced photosynthetic plasticity, and preferential accumulation of soluble sugars, starch, and NSC, indicating a functional shift toward C storage rather than active assimilation. Leaf age-dependent decline in photosynthetic capacity has been attributed to both structural senescence and feedback inhibition arising from sink limitation [29,30]. Similar contrasts in respiratory costs and photosynthetic plasticity have been described in conifer foliage, where older leaves show higher maintenance respiration and constrained light-use adjustment [31]. Nitrate was more effective than ammonium in promoting photosynthetic performance and carbohydrate accumulation, particularly in young leaves, thereby reducing photoinhibition risk. The resulting increase in C assimilation also provided sufficient C skeletons to support N assimilation across leaf ages. N addition also enhanced light reaction efficiency and intracellular biosynthesis, supporting plant growth and competitive advantage [32,33].

Meanwhile, N addition enhanced primary C backbones for N uptake, evidenced by increased N-metabolizing enzyme activities in both leaf ages (Figure 2). However, N metabolism was markedly age-dependent: old leaves displayed significantly higher activities of N-assimilating enzymes and greater free amino acid accumulation, indicating their central role in N assimilation and internal recycling. Compared with ammonium, nitrate had a stronger stimulating effect on NR and NiR activities, reinforcing its role in driving amino acid metabolism. This supports the view that younger organs prioritize nutrient acquisition while older organs concentrate on storage and transport [34].

Phytohormones modulating C allocation and N uptake also showed age-dependent patterns. In young leaves, N addition increased IAA and decreased SA, consistent with enhanced photosynthetic capacity and free amino acid accumulation (Figure 3). The pronounced increase in CK, especially under nitrate addition, suggests that CK may contribute to the maintenance of growth-related metabolic activity in young leaves under improved N supply [35,36,37]. Ammonium promoted lateral root emergence through effects on shoot-derived auxin mobility and stimulated ABA accumulation for N assimilation and stress adaptation [38,39]. In old leaves, higher concentrations of JA, ABA, and SA were maintained, with nitrate strongly amplifying age-dependent differences in SA accumulation. Although CK content in old leaves also increased under N addition, its increase was smaller than that in young leaves, suggesting that hormonal regulation in old leaves may remain more closely associated with stress acclimation and metabolic homeostasis than with active growth [40]. This is consistent with previous findings that nitrate addition significantly elevated SA content in old leaves, associated with optimizing C-N metabolism and supporting auxin-mediated growth [14]. Together, these results indicate that nitrate not only enhanced the accumulation of IAA and CK hormones in young leaves but also widened the age-dependent divergence in CK accumulation between young and old leaves [41].

### 3.2. N Form Addition Shapes C-N Metabolism Coordination Between Young and Old Leaves

N availability directly modulates photosynthetic efficiency and C fixation, while C status in turn affects N uptake and assimilation; maintaining C-N balance is critical for plant growth and productivity [42]. Young leaves exhibited enhanced photosynthetic performance and amino acid accumulation but comparatively lower NSC storage than old leaves (Figure 1). NSC, comprising soluble sugars and starch, serves as stored energy and a C substrate for biosynthesis [43,44]. The soluble sugar/starch ratio reflects NSC allocation, with starch functioning as a transient reserve to balance source activity and sink demand [45,46]. Old leaves consistently maintained higher soluble sugar, starch, and NSC levels alongside stronger N assimilation enzyme activities, indicating a coordinated role in C storage and N processing to stabilize metabolic homeostasis under variable N supply. Glutamate, arginine, and ornithine were particularly prominent in old leaves, consistent with their established roles as high-N/C-ratio compounds supporting N storage and long-distance transport in woody perennials [47].

Metabolomic analysis revealed age-dependent metabolic reprogramming under contrasting N forms. Young leaves preferentially enhanced aromatic and branched-chain amino acid biosynthesis under nitrate addition, supporting growth-related metabolism. Aromatic amino acid biosynthesis is tightly regulated to balance the C investment required for rapid growth and downstream biosynthesis [48]. In contrast, old leaves showed prominent upregulation of N-storage amino acids and secondary metabolites, particularly anthocyanins, reflecting a shift toward stress adaptation and resource conservation under nitrate rather than N deficiency per se [49]. Enhanced N assimilation induced by nitrate increased demand for reducing power, stimulating phenylpropanoid and anthocyanin biosynthesis as protective metabolic sinks; purine metabolism was coordinately down-regulated under N addition, especially in old leaves, implying accelerated nucleotide turnover and enhanced N reallocation.

Nitrate and ammonium differentially regulated these age-specific responses. Nitrate elicited more extensive metabolic reprogramming than ammonium in both leaf ages [50], a divergence rooted in the energetic and signaling properties of nitrate. Nitrate assimilation is closely coupled to the photosynthetic electron transport chain, competing for reducing power generated by light reactions [51]; this energetic competition may partly account for the reduced *P*_max_ observed in old leaves under nitrate addition. Beyond its energetic cost, nitrate acts as a systemic signaling molecule that triggers rapid transcriptional reprogramming [52] and integrates N assimilation with C skeleton supply [53], collectively driving the metabolic shift from primary growth toward secondary defense metabolism observed in old leaves. Network analysis identified aminoacyl-tRNA biosynthesis, ABC transporters, and cofactor biosynthesis as hub pathways, with amino acids and nucleotides as key candidates linking metabolic regulation to C-N physiological traits.

In summary, this study reveals a leaf age-dependent regulatory framework governing responses to contrasting N forms in Chinese fir. Young leaves prioritized photosynthetic C fixation, growth-related amino acid metabolism, and IAA- and CK-associated growth processes, while old leaves were characterized by enhanced N assimilation, C storage, and secondary metabolite accumulation. Nitrate triggered more extensive physiological and metabolic reprogramming than ammonium, strengthening the functional divergence between leaf ages. These findings advance our understanding of nutrient-use strategies in woody plant leaves under heterogeneous N environments.

## 4. Materials and Methods

### 4.1. Study Region and Experiment Design

The N addition experiment was conducted at Hongya National Forest Farm in the western Sichuan Province, China (29°38′ N, 102°58′ E), with a humid subtropical climate. The site had an average annual sunlight duration of 1006.1 h, a mean annual temperature of 16.6 °C and precipitation of 1453.5 mm. Prior to fertilization addition, the stand was a two-year-old Chinese fir plantation with a density of about 1200 trees per hectare and an average height of around 50 cm. The experiment used a randomized block design with three blocks, each containing three 15 m × 15 m plots. The basic soil properties were determined as follows: pH 5.02, organic C of 35.29 g kg^−1^, available N of 65.58 mg kg^−1^, available P of 12.65 mg kg^−1^ and available K of 51.87 mg kg^−1^. The treatments contained nitrate addition (5 g NO_3_^−^ m^−2^ year^−1^), ammonium addition (5 g NH_4_^+^ m^−2^ year^−1^) and a control (no N addition). Treatments were randomly assigned to plots within each block, with a 20 m buffer region between adjacent plots. (NH_4_)_2_SO_4_ and Ca(NO_3_)_2_ were applied to the N addition treatments, and distilled water was used for the control. Ca(NO_3_)_2_ was used as the nitrate source to avoid introducing additional potassium that might affect nutrient balance and plant physiological responses. All treatments were applied directly to the soil. The treatment lasted from April to October in 2022.

### 4.2. Foliage Sampling

At the field site, Chinese fir leaves were classified into two age groups—current year (young) and two-year-old (old) leaves—distinguished by leaf color and bud scars. The corresponding samples for treatments were as follows: young leaves (YCON) and old leaves (OCON) under control, young leaves (YAN) and old leaves (OAN) under ammonium addition, and young leaves (YNN) and old leaves (ONN) under nitrate addition. In October 2022, at least 40 leaves from each age group were randomly sampled from each branch and transported to the laboratory and stored at −80 °C for subsequent physiological, morphological and metabolomic analyses.

### 4.3. Observation of Leaf Ultrastructure Characteristics

Leaves for ultrastructure observation were randomly selected from each treatment and age class. For each biological replicate, one leaf was randomly selected from the sampled leaf pool, and a small segment from its middle portion was fixed in 3% (*v*/*v*) glutaric aldehyde in 0.2 M phosphate buffer (pH 7.2) under negative pressure in a syringe. After fixation, the samples were dehydrated in an ethanol series and embedded in Epoxy Embedding Medium (Sigma-Aldrich Chemie, Steinheim, Germany). Ultrathin sections (80 nm) were cut using a 45° diamond knife on an ultramicrotome and stained with 3% uranyl acetate and 2.7% lead citrate. The sections were then mounted on copper grids and observed under a JEM 2100 plus transmission electron microscopy (JEOL, Tokyo, Japan). Digital images were captured with an EMSIS QUEMESA TEM CCD camera (EMSIS GmbH, Münster, Germany). At least three micrographs per sample were acquired and analyzed to assess ultrastructural changes.

### 4.4. Photosynthetic Capacity Performance

Due to the small area of individual Chinese fir leaves, gas-exchange measurements for each biological replicate were conducted using a set of 4–5 intact leaves of the same age class randomly selected from the same branch. Leaf photosynthesis rate was measured using a LI-6400 portable infrared gas analyzer (LI-Cor Biosciences, Lincoln, NE, USA) to produce light response curves in descending order of 2000, 1800, 1600, 1400, 1200, 1000, 800, 600, 400, 200, 100, 50 and 0 μmol m^−2^ s^−1^. Prior to measurements, leaves were acclimated in the chamber at 25 °C, with ambient humidity and 400 μmol mol^−1^ CO_2_ for 30 min to stabilize leaf photosynthesis. The net photosynthetic rate (*P*_n_) was automatically recorded at 3 min intervals at each light level. Maximum net photosynthesis (*P*_max_) was determined from the plateau in the light response curves and the rectangular hyperbola model for *P*_max_, dark respiration rate (*R*_d_), light compensation point (*L*_CP_), light saturation point (*L*_SP_) and apparent quantum yield (AQY) [54].

### 4.5. Analysis of Leaf Physiological Functional Traits

Non-structural carbohydrates (NSCs) were determined by the phenol–sulfuric acid method [55]. For each biological replicate, the sampled leaves were pooled, cut into small pieces, and thoroughly mixed before subsampling for the following physiological and biochemical analyses. For each determination, approximately 0.01 g fresh tissue was weighed, and the exact fresh weight was recorded for subsequent calculation. Plant samples were extracted with 80% alcohol to obtain the supernatant for soluble sugar determination, followed by the extraction of residues with 1 M H_2_SO_4_ to determine starch after a water bath and centrifugation. Soluble sugar and starch were measured spectrophotometrically at 625 nm using the enthrone reagent. NSC was calculated as the sum of soluble sugar and starch concentrations. Rubisco activity was determined by monitoring NADPH oxidation at 340 nm [56]. Nitrate reductase (NR) activity and nitrite reductase (NiR) activity were determined using commercial assay kits (ARG82016 and ARG83413, Arigo Biolaboratories Corp., Shanghai, China). Glutamine synthetase (GS), glutamate synthase (GOGAT), and glutamate dehydrogenase (GDH) activities were measured using commercial assay kits (Beijing Solarbio Science & Technology Co., Ltd., Beijing, China) via spectrophotometry (GS: Cat. BC0915; GOGAT: Cat. BC0075; GDH: Cat. BC1460). Total free amino acids were extracted by ultrasonication with 5% (*w*/*v*) aqueous trifluoroacetic acid, followed by ultrafiltration and detection by high-performance liquid chromatography (HPLC) [57].

### 4.6. Determination of Phytohormone Contents

Phytohormone contents in leaf samples were determined using established methods [58]. For each determination, approximately 0.01 g fresh tissue was weighed from pooled leaf samples prepared as described above. IAA content was measured by the phenol–sulfuric acid method after extraction with 95% ethanol and quantified by spectrophotometric absorbance using a standard curve. JA content was determined by gas chromatography after extraction with acetonitrile: water (1:1, *v*/*v*) and ultrasonic treatment. ABA and CK contents were quantified using an enzyme-linked immunosorbent assay (ELISA) according to the manufacturer’s instructions. SA content was analyzed by high-performance liquid chromatography following liquid N grinding and extraction.

### 4.7. Metabolome Profiling

For metabolomic analysis, the sampled leaves within each biological replicate were pooled and processed as one composite sample. Freeze-dried biological samples were ground into fine powder using a mixer mill (MM 400, Retsch GmbH, Haan, Germany) at 30 Hz for 1.5 min. Approximately 100 mg of the powdered sample was dissolved in 1.0 mL of extraction solution (70% methanol containing 0.1 mg L^−1^ lidocaine as an internal standard). The mixture was incubated overnight at 4 °C, vortexed three times to improve extraction efficiency. After centrifugation at 10,000× *g* for 10 min, the supernatant was collected, filtered through a 0.22 μm microporous membrane, transferred into sample vials, and stored at 4 °C prior to metabolite detection. To monitor the stability and repeatability of the analytical system, quality control (QC) samples were prepared by pooling equal volumes of all individual samples and were injected at regular intervals throughout the analytical run.

Metabolite detection was performed using UPLC-MS/MS (UPLC–MS/MS; Thermo Fisher Scientific, Waltham, MA, USA), and the quantification and identification were conducted by Wuhan Metware Biotechnology Co., Ltd. (Wuhan, China). Metabolite identification was based on public databases, including MassBank, LipidMaps, mzCloud, and Kyoto Encyclopedia of Genes and Genomes (KEGG), with supplementary confirmation using Human Metabolome Database (HMDB). Principal component analysis (PCA) and partial least squares-discriminant analysis (PLS-DA) were applied to evaluate metabolite accumulation patterns using the R package ‘ropls’ (version 4.3.1). Differentially accumulated metabolites (DAMs) were identified based on variable importance in projection (VIP) values > 1 and |log_2_ fold change| > 1 and a statistically significant *p*-value < 0.05 derived from a Student’s *t*-test (adjusted by the False Discovery Rate). K-means clustering analysis in the R package ‘stats’ (version 4.3.1) was used to group DAMs into subclasses, with the optimal cluster number determined by the Calinski–Harabasz index. KEGG pathway enrichment was conducted for DAMs in each subclass to identify significantly enriched metabolic pathways. Metabolic pathway and pathway–metabolite networks were constructed using Cytoscape software (3.10.3). Network topology analysis was performed to assess pathway–metabolic interactions.

### 4.8. Statistical Analysis

One-way analysis of variance in SPSS 27.0 was used to analyze the significance of differences (*p* < 0.05) between leaf ages in the physiological data across contrasting N forms addition. Graphs were plotted using Origin 2018 software. All physiological and biochemical measurements were performed with at least three independent biological replicates. One-way analysis of variance (ANOVA) was conducted using SPSS 26.0 to test for significant differences among treatments (*p* < 0.05). The least significant difference (LSD) test was used for multiple comparisons when significant differences were detected. Graphs were plotted using Origin 2021 software.

## Data Availability

The metabolic datasets generated and analyzed during this study are available in the OMIX database under accession number OMIX015273.

## Supplementary Materials

The following supporting information can be downloaded at: https://www.mdpi.com/article/10.3390/ijms27093789/s1.

## Abbreviations

The following abbreviations are used in this manuscript:

NSC	Non-structural carbohydrates
*P*_max_	Maximum net photosynthetic rate
*P*_n_	Net photosynthetic rate
*L*_SP_	Light saturation point
*L*_CP_	Light compensation point
*R*_d_	Dark respiration rate
AQY	Apparent quantum yield
IAA	Indole-3-acetic acid
ABA	Abscisic acid
SA	Salicylic acid
JA	Jasmonic acid
CK	Cytokinin
NR	Nitrate reductase
NiR	Nitrite reductase
GS	Glutamine synthetase
GOGAT	Glutamate synthase
GDH	Glutamate dehydrogenase
Rubisco	Ribulose-1,5-bisphosphate carboxylase/oxygenase
DAMs	Differentially accumulated metabolites
VIP	Variable importance in projection
PCA	Principal component analysis
PLS-DA	Partial least squares-discriminant analysis
KEGG	Kyoto Encyclopedia of Genes and Genomes
HMDB	Human Metabolome Database
UPLC-MS/MS	Ultra-performance liquid chromatography tandem mass spectrometry
