# Supplementary material for: Leaf Ontogeny Shapes Divergent Physiological and Metabolic Responses to Contrasting Nitrogen Forms in Chinese Fir (Cunninghamia lanceolata (Lamb.) Hook)
Source: Int J Mol Sci. 2026 Apr 24;27(9):3789. doi: 10.3390/ijms27093789 (PMC13163334; doi:10.3390/ijms27093789)
Supplement: Supplementary file 1 [file ijms-27-03789-s001.zip › ijms-4227240-supplementary.pdf]

Figure S1. KEGG pathway enrichment analysis of differentially accumulated metabolites (DAMs) in pairwise comparisons. (A–F) Significantly enriched KEGG pathways for the pairwise comparisons. The rich factor represents the ratio of the number of metabolites mapped to a given pathway to the total number of annotated metabolites. Bubble size indicates the number of metabolites enriched in each pathway, and bubble color reflects the enrichment significance level. No significantly enriched KEGG pathways were detected for the YCON vs OCON comparison. YCON, young leaves under control conditions without N addition; YAN, young leaves treated with NH_4^+–N addition; YNN, young leaves treated with NO_3^-–N addition; OCON, old leaves under control conditions without N addition; OAN, old leaves treated with NH_4^+–N addition; ONN, old leaves treated with NO_3^-–N addition.

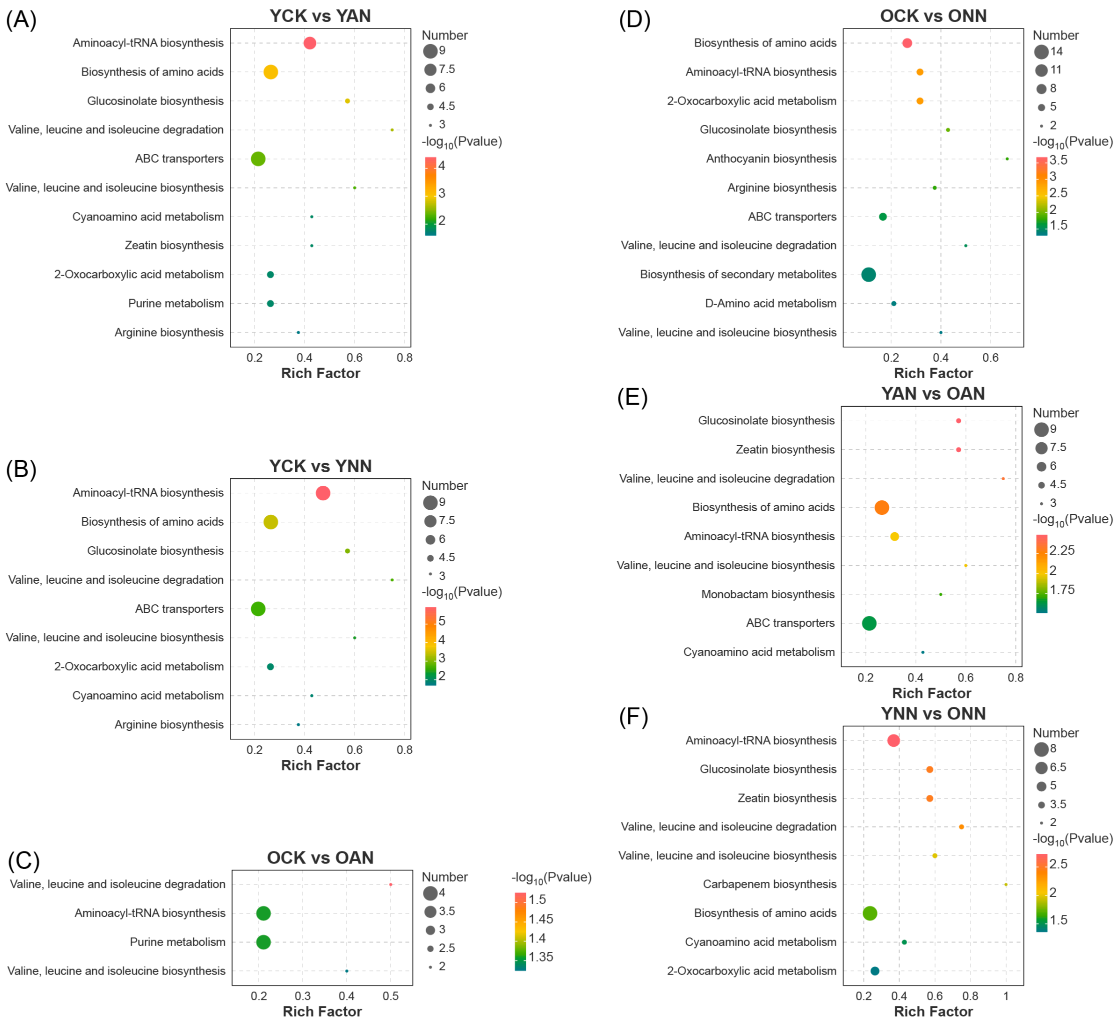

Table S1. Specific DAMs between ammonium and nitrate additions in different leaves.

Comparisons	Compounds	log_2FC	VIP
YCON vs YAN	**Fatty acids**		
	LysoPC 18:2	1.73	1.24
	**Flavonoids**		
	Tricin 4'-O-(syringyl alcohol) ether 5-O-hexoside	-2.07	2.39
	Procyanidin B2	-1.01	3.10
	Procyanidin A1	3.14	1.13
	Procyanidin A2	3.48	1.29
	**Organic acids**		
	Gallic acid O-Hexoside	-2.72	1.10
	Caffeic acid O-glucoside	-1.89	1.02
	Kynurenic acid	1.21	1.94
	**Plant hormones**		
	Salicylic acid O-glucoside	-1.27	1.70
	**Others**		
	Pyridoxine O-glucoside	-1.14	2.53
	Pyridoxine	1.08	1.28
YCON vs YNN	**Flavonoids**		
	2'-Hydroxygenistein	1.17	1.73
	**Organic acids**		
	5-O-p-coumaroyl quinic acid O-hexoside	-1.39	4.03
	**Plant hormones**		
	Abscisic acid	1.04	1.31
	**Others**		
	N', N''-disinapoylspermidine	-1.67	1.88
OCON vs OAN	**Amino acids**		
	3-Hydroxykynurenine	2.37	1.31

	**Flavonoids**		
	Prunetin	-1.71	1.11
	Acacetin	-1.60	1.09
	Chrysoeriol C-hexosyl-O-rhamnoside	4.97	2.13
	Chrysoeriol 7-O-rutinoside	5.07	2.18
	Gallocatechin-catechin	5.37	1.19
	**Organic acids**		
	3-O-Feruloyl quinic acid glucoside	-2.09	1.07
	5-O-Feruloyl quinic acid glucoside	-2.09	1.15
	1-O-Caffeoyl quinic acid	-1.44	6.50
	**Plant hormones**		
	Indole	-1.31	1.13
	**Others**		
	Putrescine	-1.52	1.03
	4-Pyridoxate	-1.02	1.25
OCON vs ONN	**Amino acids**		
	L-Ornithine	6.44	1.13
	H-HomoArg-OH	6.68	1.01
	**Flavonoids**		
	Prunin	1.13	1.36
	Tricin 7-O-hexoside	1.21	1.35
	Pelargonin	2.30	1.03
	Keracyanin	3.00	1.28
	Epigallocatechol	3.75	2.36
	(+)-Gallocatechin	3.82	2.43
	Gallocatechin-gallocatechin	20.16	1.30
	**Organic acids**		
	D-Malic acid	-1.77	5.64

Table S2. Specific DAMs between young and old leaves under N addition.

Comparisons	Compounds	log_2FC	VIP
YCON vs OCON	**Flavonoids**		
	Prunin	-1.35	1.61
	Luteoloside	-1.03	3.01
	Astragalin	-1.03	3.07
	Chrysoeriol 5-O-hexoside	3.23	2.10
	**Nucleotides and Derivatives**		
	3'-Adenylic acid	-1.17	1.45
	Adenosine O-ribose	2.36	2.57
	**Organic acids**		
	Cis-Aconitic acid	-2.34	1.18
	Coniferyl aldehyde	-1.84	1.04
	Caffeic acid O-glucoside	-1.49	1.28
	2–furanoic acid	-1.32	1.60
	Citric acid	-1.14	3.80
	3-O-p-coumaroyl quinic acid O-hexoside	-1.06	4.75
	Kynurenic acid	1.20	2.64
	Sinapoyl malate	1.26	1.00
	**Plant hormones**		
	Kinetin 9-riboside	1.95	1.55
	5-Methoxyindoleacetic acid	2.72	1.05
	Indole	2.76	1.20
	**Others**		
	6-Methoxy-7,8-DihydroxyCoumarin	-1.34	1.62
	4-Pyridoxate	1.06	1.09
YAN vs OAN	**Amino acids**		
	L-Citrulline	-4.63	2.74
	L-Arginine	-1.69	1.97
	**Fatty acids**		
	MAG (18:3) isomer3	-1.61	1.01
	LysoPC 18:2	-1.60	1.16
	LysoPC 18:2 (2n isomer)	-1.21	1.07

	**Flavonoids**		
	Procyanidin A1	-20.51	1.16
	Procyanidin A2	-2.03	1.10
	**Others**		
	4-Oxoretinol	1.62	1.05
	**Amino acids**		
	L-Glutamate	-1.16	2.74
	**Fatty acids**		
	LysoPC 16:0	-1.46	3.04
	LysoPC 18:1 (2n isomer)	-1.23	1.73
	**Flavonoids**		
YNN vs ONN	Tricin 7-O-hexoside	2.27	1.21
	Epigallocatechol	4.90	1.83
	(+)-Gallocatechin	4.95	1.88
	Keracyanin	5.63	1.04
	**Nucleotides and Derivatives**		
	6-Methylmercaptopurine	-1.46	1.67
	**Others**		
	10-Formyltetrahydrofolate	-2.48	1.55
